# A feasibility study of robot-assisted percutaneous reduction and fixation technique for treating posterolateral depression tibial plateau fractures

**DOI:** 10.1038/s41598-023-49624-x

**Published:** 2023-12-12

**Authors:** Yao Lu, YiBo Xu, Cheng Ren, Zhong Li, Kun Zhang, Qiang Huang, Teng Ma

**Affiliations:** Department of Orthopedics, Hong Hui Hospitalospital, Xi’an Jiaotong University, Xi’an, 710054 Shaanxi China

**Keywords:** Trauma, Bone

## Abstract

Posterolateral (PL)-depression fractures of the tibial plateau are difficult to manage. The aim of this study was: (1) to present our experience with a novel technique of robot-assisted percutaneous reduction and fixation and (2) to compare it with the traditional percutaneous screw osteosynthesis (PSO) technique for the treatment of PL-depression tibial plateau fractures. The clinical data of patients with PL-depression tibial plateau fractures treated by robot-assisted percutaneous reduction and fixation technique and traditional PSO technique from January 2017 to January 2021 were retrospectively analyzed. Among them, there were 18 cases in the robot-assisted group (RA group) and 23 cases in the PSO group. All fractures were unilateral, closed and fresh PL-depression fractures of the tibial plateau. Patients in the RA group were treated by robot assisted reduction and minimally invasive absorbable screw fixation. The PSO group was treated by closed reduction and percutaneous absorbable screw osteosynthesis. The incision length, operation time, intraoperative blood loss, fluoroscopic times, inpatient time, weight training time and postoperative complications of the two groups were statistically analyzed. The Rasmussen radiological score was used to assess the reduction quality after operation while the Rasmussen functional score was used to evaluate knee joint functions at one year postoperatively. All patients were followed for at least one year. There was no significant difference in demographic information between the two groups (*p* > 0.05). Compared with the PSO group, the RA group showed less fluoroscopic times and better Rasmussen radiological and functional scores (*p* < 0.05). Besides, there was no significant difference in the incidence of postoperative complications between the two groups (*p* > 0.05). The novel robot-assisted percutaneous reduction and fixation technique had the characteristics of less radiation, accurate reduction and fixation. It could accelerate the rehabilitation of patients with PL-depression fractures of the tibial plateau and enable patients to obtain good joint functions.

## Introduction

Posterolateral depression fractures of the tibial plateau are a special fracture type of tibial plateau. This type of fracture is usually caused by knee flexion and valgus combined with axial violence^1^. It belongs to intra-articular fracture. If the depression is more than 2 mm, anatomical reduction and strong fixation is the first treatment choice^2^. However, there are important structures such as the common peroneal nerve, tibial nerve and popliteal artery behind the tibial plateau, and lateral collateral ligament (LCL) on the lateral side, so it is difficult to expose the PL-depression fragment directly. These unique anatomical factors make the treatment of the PL-depression tibial plateau fracture difficult.

Scholars have tried different surgical approaches and assisted reduction tools to treat the PL-depression tibial plateau fractures. Currently, the surgical approaches reported in the literature for this fracture include different anterolateral approaches, posterolateral approaches and the fibular osteotomy approach, etc.^3–8^. Although the anterior or anterolateral approaches could avoid anatomy of nerve and vascular structures, the reduction and fixation of simple PL-column tibial plateau fractures are usually indirect, which is not conducive to early functional exercises. The PL tibial plateau could be well exposed through the fibular head osteotomy approach, but the incidence of complications is high, including the common peroneal nerve injury, lateral instability of the knee joint, and nonunion of the fibula^4,8^. Similarly, surgeons face thick soft tissues and the risk of damage to nerve and vessels via the posterolateral approach. Closed reduction or minimally invasive reduction under X-ray guidance is less traumatic, but the effects of articular cartilage reduction are not accurate^9^. Arthroscopy can directly observe the situation in the joint cavity, handle ligament injuries and meniscus injuries, and monitor the reduction of intra-articular fractures. However, its role in reduction and fixation is limited^10,11^.

In recent years, the application of computer navigation and robot-assisted internal fixation technique has gradually developed in orthopedic surgeries. Some scholars have applied this novel technique to treat pelvic, acetabular, femoral neck and spinal fractures and achieved satisfactory results^12–14^. Robot navigation technique has significant advantages in reducing trauma, accurate positioning, reducing X-ray radiation exposure and complications^15,16^. Nevertheless, there is no reports on the clinical study of robot-assisted percutaneous reduction and internal fixation for the treatment of PL-depression tibial plateau fractures. Our team has creatively applied this novel technique to patients with this type of fractures and achieved good results.

The aim of this study was: (1) to present our experience with this novel technique of robot-assisted percutaneous reduction and fixation and (2) to compare it with traditional percutaneous screw osteosynthesis. technique for the treatment of PL-depression tibial plateau fractures. We retrospectively collected relevant clinical data and evaluated the efficacy.

## Materials and methods

### Patients

The written informed consent was obtained from all patients. All methods were carried out following relevant guidelines and regulations. The ethics committee of Xi’an Hong Hui hospital has approved this study. The clinical data of patients with PL-depression tibial plateau fractures treated in Xi’an Hong Hui hospital from January 2018 to January 2021 were retrospectively analyzed. Forty-one cases were included. Among them, 18 cases were treated by the robot-assisted percutaneous reduction and fixation technique (RA group) while 23 cases by the closed reduction and percutaneous screw osteosynthesis technique (PSO group). The inclusion criteria were: (i) Patients over 18 years; (ii) Patients meeting the diagnostic criteria of PL-depression tibial plateau fractures; (iii) The fractures were closed and fresh; (iv) Patients were treated by the RA or PSO technique and followed for at least one year; (v) Patients with complete medical records. The exclusion criteria were: (i) Poly-trauma patients; (ii) Patients with nerve and vascular injuries; (iii) Patients with open fractures; (iv) Patients with pathological fractures; (v) Patients with history of ipsilateral knee arthritis. In terms of fracture classification, these fractures all were PL-depression tibial plateau fractures. Only or mainly the PL-column of the tibial plateau was involved. According to the depression depth, these fractures were further divided into two types, including depression depth within 2–5 mm and deeper than 5 mm.

### Preoperative treatment

All patients underwent anteroposterior (AP) and lateral X-ray films, CT scan and three-dimensional reconstruction before operation. These patients also underwent all other necessary preoperative examinations. Surgical contraindications were excluded, and medical diseases were actively adjusted to meet the surgical requirements. Before operation, patients were given ice compress, detumescence and pain-relief management. Low molecular heparin was given to prevent deep venous thrombosis (DVT) of lower limbs.

The robot is Ti-robot (TINAVI Medical Technologies, Beijing, China), which is the third generation of TINAVI orthopedic surgery robot. It consists of a main control workstation, surgical planning and control software, a manipulator, an optical tracking system and matching tools. The robot navigation arm owns six degrees of freedom. The optical tracking system is composed of an infrared based binocular camera with a positioning accuracy of less than 0.3 mm. By closely tracking the tracer of the injured limb and the robot arm, the spatial position of the robot arm could be accurately located in real time. The surgical planning and control software is used for the collection and processing of image data, the design of surgical path and the driving of robot arm movement, and real-time control and accurate adjustment.

### Surgical procedures of the RA group

**Figure 1:**
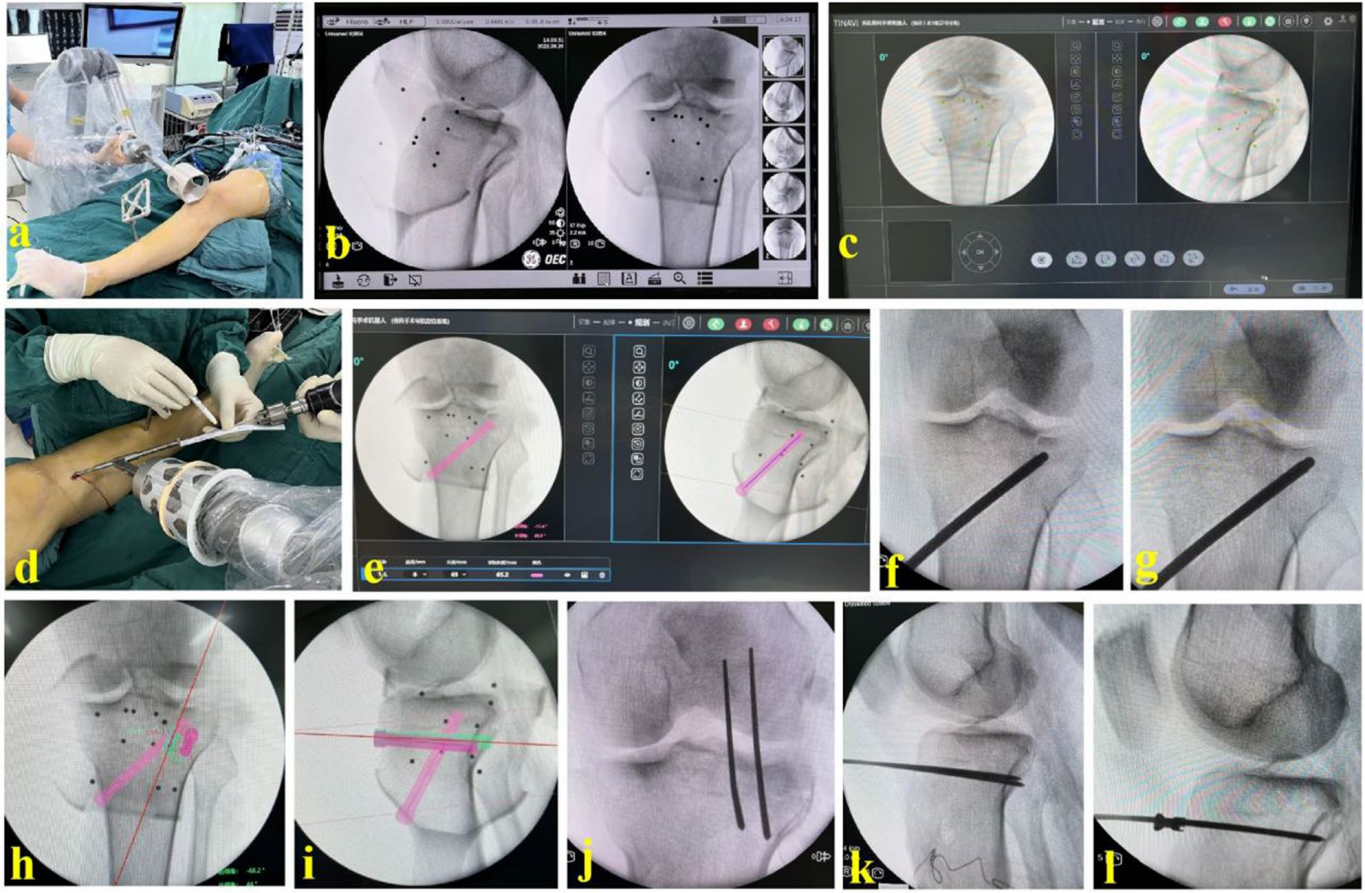
The intraoperative setting up and application procedures of the orthopedic robot system for the PL-depression tibia plateau fractures. (**a**,**b**) The robot system was set up and standard AP and lateral X-ray images of the tibial plateau were achieved; (**c**) The standard AP and lateral X-ray images were imported into the planning and control software; (**d**–**g**) The reduction path was planned on the planning and control software, and the PL-depression fragment was successfully reduced by a reduction bone rod via robot navigation; (**h**–**l**) The absorbable screw channels were planned on the planning and control software, and two absorbable screws were inserted via robot navigation.

**Figure 2:**
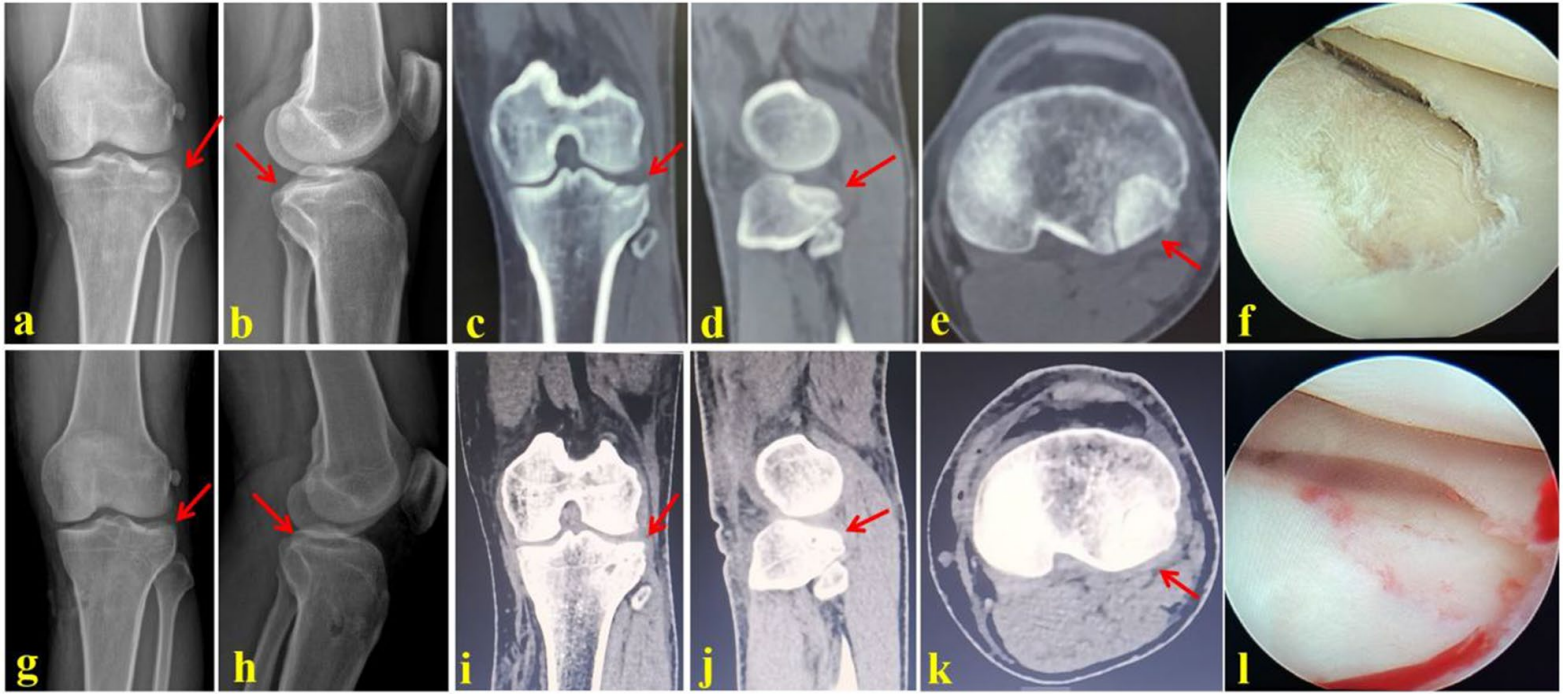
A 37-year-old male suffered from the PL-depression tibial plateau fracture and was successfully treated by the RA technique. (**a**,**b**) AP and lateral X-ray images of the injured tibial plateau; (**c**–**e**) Coronal, horizontal and sagittal CT scan images before operation; (**f**) Arthroscopic image of the PL-depression fragment before operation; (**g**,**h**) AP and lateral X-ray images of the tibial plateau after operation; (**i**–**k**) Coronal, horizontal and sagittal CT scan images after operation; (**l**) Arthroscopic image of the PL-depression fragment after operation. Red arrows indicate the PL-depression fragment of the tibial plateau.

(i) The depression fragment of articular surface was observed through arthroscopy first. The robot system was prepared. The position of each component of the robot was adjusted to ensure that the optical tracking system and the robot arm were in a reasonable position. An optical tracer was inserted in the middle tibia of the injured limb. Standard AP and lateral X-ray images of the tibial plateau were taken (Fig. 1a,b). The AP and lateral images were imported to the planning and control software (Fig. 1c).
(ii) The depression position was accurately located and the reduction channel was planned (Fig. 1d,e). The robot arm automatically moved to the designated position according to the planned surgical plan. During the movement of the manipulator, the planning control system combined with the optical tracking system could monitor and adjust the movement trajectory of the manipulator in real time to ensure the stable and accurate spatial positioning of the manipulator.
(iii) The guide pin was inserted and the reduction channel was established (Fig. 1f). A wound of 2 cm was cut at the entry point of the guide pin. Blunt separation was performed until bone surface was exposed. Fenestration was made in the anteromedial bone cortex of the tibia. The hollow shaft was inserted along the guide pin to expand the reduction channel for accommodating the reduction bone rod.
(iv) The reduction bone rod was inserted to reduce the PL-depression fragment of the tibial plateau (Fig. 1f,g). Intraoperative fluoroscopy was used to evaluate the reduction of the PL-depression fragment and whether the knee joint surface was restored. After removing the reduction bone rod, artificial bones (allografts, Orui Biomaterials Co., Ltd, Shanxi, China) were fetched to fill the reduction channel and support the reduced articular surface.
(v) The absorbable screw paths of 2–3 with appropriate length were designed on the planning control software (Fig. 1h,i). The path was from the inside down to the outside up or from the front to the back. In order to provide stable internal fixation and support for the PL-depression fragment and artificial bones, 2–3 hollow absorbable screws (3.5 mm) were inserted percutaneously with the assistance of robot navigation (Fig. 1j–l). The quality of articular surface reduction was confirmed by arthroscopy again. A typical case of the RA group is shown in Fig. 2.

### Surgical procedures of the PSO group

**Figure 3:**
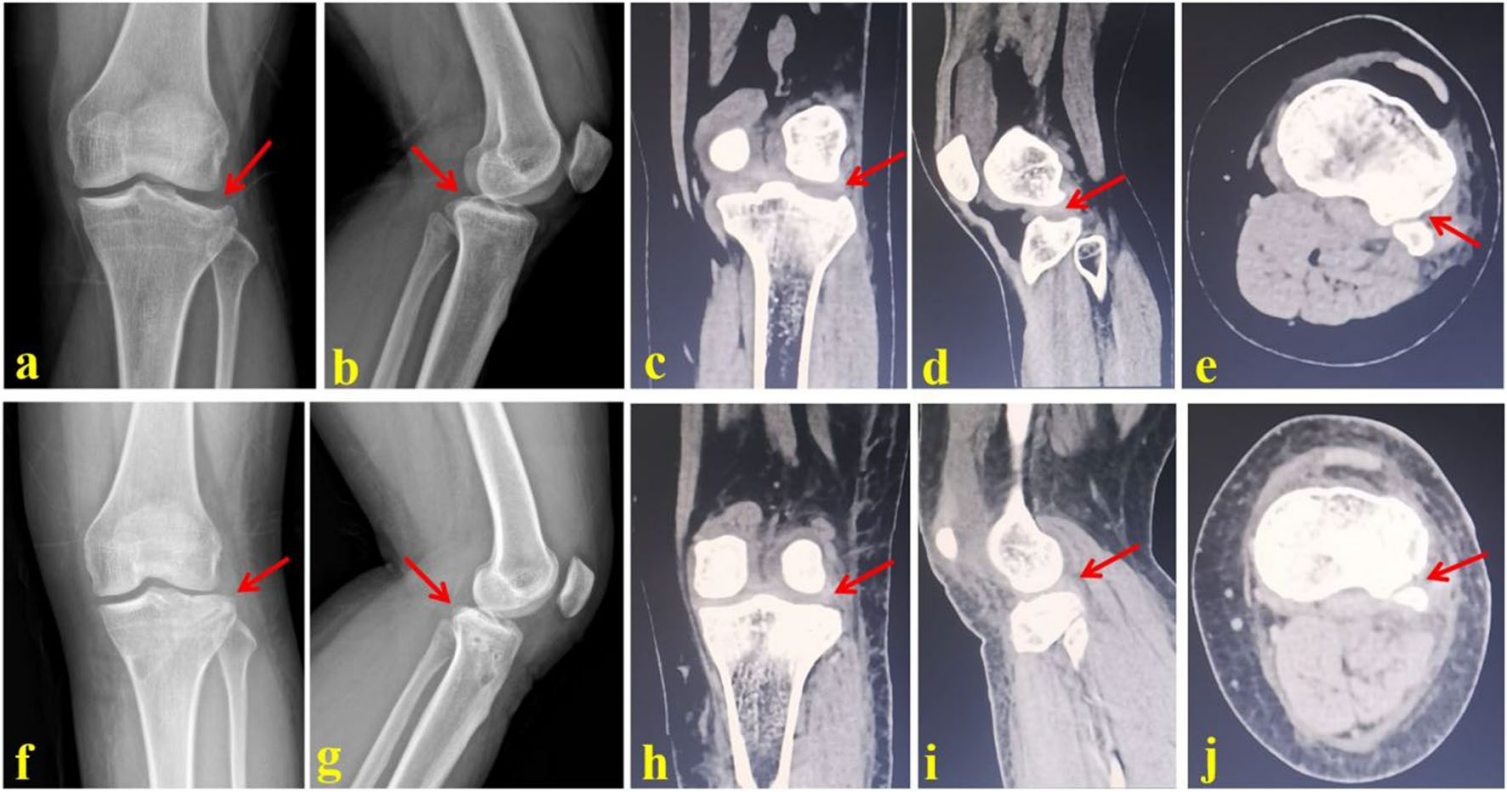
A 35-year-old female suffered from the PL-depression tibial plateau fracture and was successfully treated by the PSO technique. (**a**,**b**): AP and lateral X-ray images of the injured tibial plateau; (**c**–**e**) Coronal, horizontal and sagittal CT scan images before operation; (**f**,**g**) AP and lateral X-ray images of the tibial plateau after operation; (**h**–**j**) Coronal, horizontal and sagittal CT scan images after operation. Red arrows indicate the PL-depression fragment of the tibial plateau.

A small incision of 2 cm at the anteromedial side of the proximal calf was taken. Fenestration was performed in this incision. Then, the reduction channel was established under the C-arm image intensifier The PL-depression fragment was jacked up with the reduction rod. Fluoroscopy was taken to confirm the reduction of articular surface. Artificial bones were inserted into the bone defect site. 2–3 hollow absorbable screws (3.5 mm) were percutaneously inserted to support the posterolateral fragment. The screw length and direction were confirmed under the C-arm image intensifier. A typical case of the PSO group is shown in Fig. 3.

### Postoperative treatment

Routine anti-swelling, pain-relief and anti-infection treatment were given after operation. Isometric contraction of the quadriceps femoris, flexion and extension of the ankle joint and toes started on the first day after operation. According to the reexamination, patients were usually allowed to carry out partial weight-bearing activities 4–6 weeks after operation, and were allowed to carry out full weight-bearing activities after the fracture completely healed. These patients were followed once a month in the first three months and once every two or three months thereafter. AP, lateral X-ray images and CT scan of the injured limb were performed during follow-up periods, and functional exercises were guided.

### Main observation indicators

The incision length, operation time, intraoperative blood loss, fluoroscopic times, inpatient time, weight training time and postoperative complications of the two groups were recorded. The intraoperative blood was wiped by large and small gauze blocks. When the large gauze is completely soaked with blood, the bleeding volume is about 50 ml. When the small gauze is completely soaked with blood, the bleeding volume is about 10 ml. If the gauze is not fully saturated with blood, the blood loss is estimated according to the infiltration area. The blood volume in the gauze blocks are added together, which is the final bleeding during the operation. The Rasmussen radiological score was used to assess the reduction quality after operation, comprising depression (6 points), condylar widening (6 points), and angulation (6 points), with a total score of 18 points^17^. According to the score, it was rated as excellent (18 points), good (12–17 points), fair (6–11 points), and poor (< 6 points). In addition, Rasmussen functional score was used to evaluate knee joint functions at one year postoperatively, including pain (6 points), walking capacity (6 points), extension (6 points), range of motion (6 points), and stability (6 points), with a total score of 30 points^17^. Based on the functional score, it was rated as excellent (≥ 27 points), good (20–26 points), fair (10–19 points), and poor (6–9 points).

### Statistical processing

SPSS 24.0 software was used to process data. Measurement data were expressed as mean ± standard deviation by unpaired t test. Count data were analyzed using χ^2^ test. *P* < 0.05 was defined as statistically significant.

### Consent to participate/consent to publish

All patients or their family members have signed the informed consent before surgery and provided the consent to publish and report individual clinical data.

## Results

### Comparison of demographics of the two groups

As shown in Table 1, the mean age was 38 ± 9 years in the RA group vs. 36 ± 11 years in the PSO group, respectively. There were 11 males and 7 females in the RA group while 15 males and 8 females in the PSO group. Nine cases suffered from traffic accident, 5 from falling height, and 4 cases from others for the RA group. In the PSO group, 11 cases encountered traffic accident, 7 due to falling height, and 5 for others. With respect to classification, there were 15 cases (depression depth within 2–5 mm) and 3 cases (deeper than 5 mm) in the RA group while 17 cases (2–5 mm) and 6 cases (> 5 mm) in the PSO group, respectively. The mean body mass index (BMI) was 25.7 ± 2.8 kg/m^2^ in the RA group vs. 24.4 ± 3.5 kg/m^2^ in the PSO group, respectively; the mean time from injury to operation was 3.7 ± 1.3 days vs. 3.5 ± 1.6 days, respectively; and the average follow-up time was 21 ± 5 months vs. 23 ± 4 months, respectively. There was no significant difference for the demographics between the two groups (*p* > 0.05).

**Table 1:** Comparison of demographic information between the two groups.

Variable	RA group (n = 18)	PSO group (n = 23)	*p* value
Age (year)	38 ± 9	36 ± 11	0.526
Gender (M/F)	11/7	15/8	0.786
Etiology			0.983
Traffic accident	9	11	
Falling height	5	7	
Others	4	5	
Classification			0.732
Depression depth (2–5 mm)	15	17	
Depression depth (> 5 mm)	3	6	
BMI (kg/m^2^)	25.7 ± 2.8	24.4 ± 3.5	0.194
Time from injury to operation (d)	3.7 ± 1.3	3.5 ± 1.6	0.661
Follow-up (month)	21 ± 5	23 ± 4	0.176

RA stands for robot-assisted. PSO stands for percutaneous screw osteosynthesis. BMI stands for body mass index.

### Clinical evaluation of the two groups

Table 2 showed the clinical evaluation of the two groups. The mean incision length was 5.6 ± 1.8 cm vs. 6.0 ± 2.1 cm in the RA and PSO groups (*p* > 0.05). For the intraoperative blood loss, the RA group was 29 ± 11 ml while the PSO group was 36 ± 15 ml, respectively (*p* > 0.05). The mean operation time was 85 ± 14 min in the RA group vs. 93 ± 22 min in the PSO group (*p* > 0.05). The fluoroscopic times was 14 ± 3 vs. 22 ± 5 for the RA and PSO patients, with significant difference between the two groups (*p* < 0.05). For the inpatient time, the RA group was 4.3 ± 1.1 days while the PSO group was 4.6 ± 1.3 days (*p* > 0.05). The mean weight training time was 3.2 ± 0.5 months vs. 3.3 ± 0.4 months for the RA and PSO patients (*p* > 0.05). For the Rasmussen radiological score, it was excellent in 16 cases and good in two cases of the RA group while excellent in 12 patients, good in 8 patients and fair in 3 patients of the PSO group, and there was significant difference between the two groups (*p* < 0.05). In terms of the Rasmussen functional score, it was excellent in 14 cases, good in 3 cases and fair in 1 case for the RA group while excellent in 9 patients, good in 9 patients and fair in 5 patients for the PSO group, with significant difference between the two groups (*p* < 0.05).

**Table 2:** Clinical evaluation of the two groups.

Variable	RA group (n = 18)	PSO group (n = 23)	*p* value
Incision length (cm)	5.6 ± 1.8	6.0 ± 2.1	0.516
Blood loss (ml)	29 ± 11	36 ± 15	0.093
Operation time (min)	85 ± 14	93 ± 22	0.165
Fluoroscopic times	14 ± 3	22 ± 5	*p* < 0.001
Inpatient time (d)	4.3 ± 1.1	4.6 ± 1.3	0.429
Weight training time (month)	3.2 ± 0.5	3.3 ± 0.4	0.494
Rasmussen radiological score			0.036
Excellent	16	12	
Good	2	8	
Fair	0	3	
Rasmussen functional score			0.044
Excellent	14	9	
Good	3	9	
Fair	1	5	

### Comparison of complications of the two groups

As shown in Table 3, there was 1 patient suffered from delayed union, 2 for reduction loss, and 1 for internal fixation failure in the RA group. However, in the PSO group, 9 complications occurred, including 1 case for incision infection, 2 for delayed union, 3 for reduction loss, 2 for internal fixation failure, and 1 case for DVT. The difference of complication incidence between the two groups was not significant (*p* > 0.05). Patients encountered incision infection were given debridement and antibiotics. Those suffered from delayed union were required not to bear load until union was achieved, so as to prevent the fracture fragment from depression again or even nonunion. All patients ultimately achieved fracture union. Patients with reduction loss underwent a revision surgery. Those encountered DVT were treated with immobilization and thrombolysis.

**Table 3:** Comparison of complications of the two groups.

Variable	RA group (n = 18)	PSO group (n = 23)	*p* value
Incision infection n (%)	0 (0%)	1 (4.3%)	–
Delayed union n (%)	1 (5.6%)	2 (8.7%)	0.825
Reduction loss n (%)	2 (11.1%)	3 (13.0%)	0.769
Internal fixation failure n (%)	1 (5.6%)	2 (8.7%)	0.825
DVT n (%)	0 (0%)	1 (4.3%)	–

DVT stands for deep vein thrombosis.

## Discussion

Since the four column classification of the tibial plateau was proposed, it has been widely recognized by the academic community^18^. The PL-depression fracture of the tibial plateau, which was sometimes missed before, has received more attention from scholars. As a type of intra-articular fractures, it has a great impact on the patient’s joint functions. If it is not properly treated, it will result in movement disorders, obvious chronic pain, and accelerating the degeneration of articular cartilage which seriously affects patient’s life quality. Active surgical treatment is advocated for such fractures with obvious depression (> 2 mm). The principle of surgical treatment is to fully restore the flatness of the articular surface, protect the ligaments and bony stable structures around the joint, and give strong internal fixation, and prevent the depression again, so as to facilitate the functional recovery of the injured limb^2,19^.

There are still great controversies among scholars about the specific surgical approaches and fixation schemes for the PL-depression tibial plateau fractures. In the traditional anterolateral approach, the posterior side is exposed to the anterior side of the fibular head at most, and only the anterior part of the PL-depression fragment can be exposed. The exposure is insufficient, which brings inconvenience to the reduction of the fracture and the insertion of a plate^7,15,16^. Although the PL-depression fragment could be reduced and fixed satisfactorily through the fibular osteotomy approach, the common peroneal nerve needs to be dissected and the fibular head needs to be cut and fixed during the operation. The operation is complicated and traumatic, and may affect the micro motion joint of the upper tibiofibular joint^4,8^. Fang et al. compared the intraarticular osteotomy approach with the anteromedial “window” osteotomy approach for PL-depression tibial plateau fractures, and demonstrated that the intraarticular osteotomy could obtain satisfactory clinical results for such patients^20^. Durigan et al. introduced a lateral femoral epicondyle osteotomy approach for the treatment of PL-depression tibial plateau fractures and found that this approach allowed direct reduction and stable fixation of the PL fragment without functional impairment^21^. Yet, although the intraarticular and epicondyle osteotomy approaches could provide direct vision of the PL fragment, the surgical trauma is large. Therefore, no matter what surgical approach is adopted, it is impossible to take into account that it is not only convenient for fracture reduction under direct vision, but also could reduce trauma and the risk of injury to important structures such as vessels, nerves and ligaments.

With the rapid development of digital orthopaedics and intelligent robots, the precise and minimally invasive treatment of fractures has become possible. At present, robot-assisted technique has been successfully applied to minimally invasive surgery of acetabulum, pelvis, spine, and femoral neck, which has significant advantages over traditional surgeries^12–14,22^. The robot surgical location and navigation system our team used is produced by Beijing TINAVI Medical Technologies, and developed independently in China and recognized internationally. Du et al. reported that orthopedic robot-assisted treatment of unstable pelvic fractures was minimally invasive and feasible in a retrospective analysis of 17 cases^23^. Liu et al. found that robot-aided minimally invasive lumbopelvic fixation for traumatic spinopelvic dissociation was a safe and feasible option and the advantages included less intraoperative blood loss, less radiation damage, less hospitalization time, and better functional outcome^22^. Our team tried to apply the robot-assisted technique in the PL-depression fractures of the tibial plateau. Based on our results, compared with the PSO technique, the RA technique showed less radiation exposure, and better reduction effects and functional recovery. Our results were similar to the above results in previous literature. RA technique and PSO technique are both minimally invasive reduction and fixation technique. In PSO group, the minimally invasive reduction through the medial metaphyseal window is regularly applied. However, approaching the PL-depression fragment through the metaphyseal window is not always easy, due to the difficulty in aiming and trajectory. This may be the reason for the high radiation exposure in the PSO group. Conversely, RA group could avoid repeated fluoroscopy via robot navigation and precise positioning. Furthermore, navigation and positioning also ensure better reduction and fixation quality. This is similar to adding an "eye" to traditional closed reduction technique. When surgeons perform closed reduction and fixation, they no longer rely solely on experience, but rather on the combination of experience and advanced navigation and positioning system. This may be the main reason why the clinical efficacy of the RA group was superior to that of the PSO group.

The safety and accuracy of robot navigation surgery are guaranteed by several aspects. The core components of the robot system are a binocular camera based on infrared ray and a six axis robot arm, and the bi plane positioning algorithm is used to achieve real-time accurate positioning in space. In addition, according to the surgical planning, the robot arm will automatically move to the planned position. During its movement, the binocular camera can monitor the position of the robot arm in real time through the tracer on the robot arm and transmit the feedback information to the control system. Moreover, in the process of guide pin insertion, the control screen dynamically displays two different trajectory graphs representing the actual trajectory and the planned trajectory. In case of deviation from the pre planned path, the surgeon could adjust the two trajectories to make them approximately coincide with each other through the operation interface, so as to correct minor errors. The literature shows that the positioning error of the robot system is less than 1 mm^14^. The control system could accurately plan the insertion path and the most appropriate depth of the reduction bone rod. During the insertion of an absorbable screw, the optimal direction and length of the screw could be individualized according to the internal fixation principle, so that it could not only achieve the strongest and stable effects but also not pass through the contralateral cortex or enter the joint cavity. Based on the high-quality reduction of the PL-depression fracture and the high-precision insertion of absorbable screws under the robot navigation, it ensures that the fracture fragment is closer to the anatomical reduction, and reduces the surgical injuries as much as possible, which is conducive to early functional exercises. In this study, radiation exposure and postoperative Rasmussen radiological and functional scores of the RA group were better than those of the PSO group, which also confirms the above points.

There are still several deficiencies in this study. During the operation, there shall be no shield between the binocular camera and the tracer of the robot arm. When the robot arm moves to the planned position, the camera may not be able to directly look at the tracer of the robot arm due to the space angle. At this time, the camera position must be manually adjusted to make the tracer within its visual range, which may cause problems such as the extension of the operation time. Due to the limited marketing time of the robot system, more studies with large sample size and long follow-up period are still needed to prove it.

## Conclusion

The novel robot-assisted percutaneous reduction and fixation technique had the characteristics of less radiation exposure, accurate reduction and fixation. It could accelerate the rehabilitation of patients with PL-depression fractures of the tibial plateau and enable patients to obtain good joint functions.

## Data Availability

The datasets analyzed during the current study are available from the corresponding author upon reasonable request.
